# Supplementary material for: SOCS3 Protein Mediates the Therapeutic Efficacy of Mesenchymal Stem Cells against Acute Lung Injury
Source: Int J Mol Sci. 2023 May 4;24(9):8256. doi: 10.3390/ijms24098256 (PMC10179427; doi:10.3390/ijms24098256)
Supplement: Supplementary file 1 [file ijms-24-08256-s001.zip › ijms-2347979-supplementary.pdf]

Supplementary materials

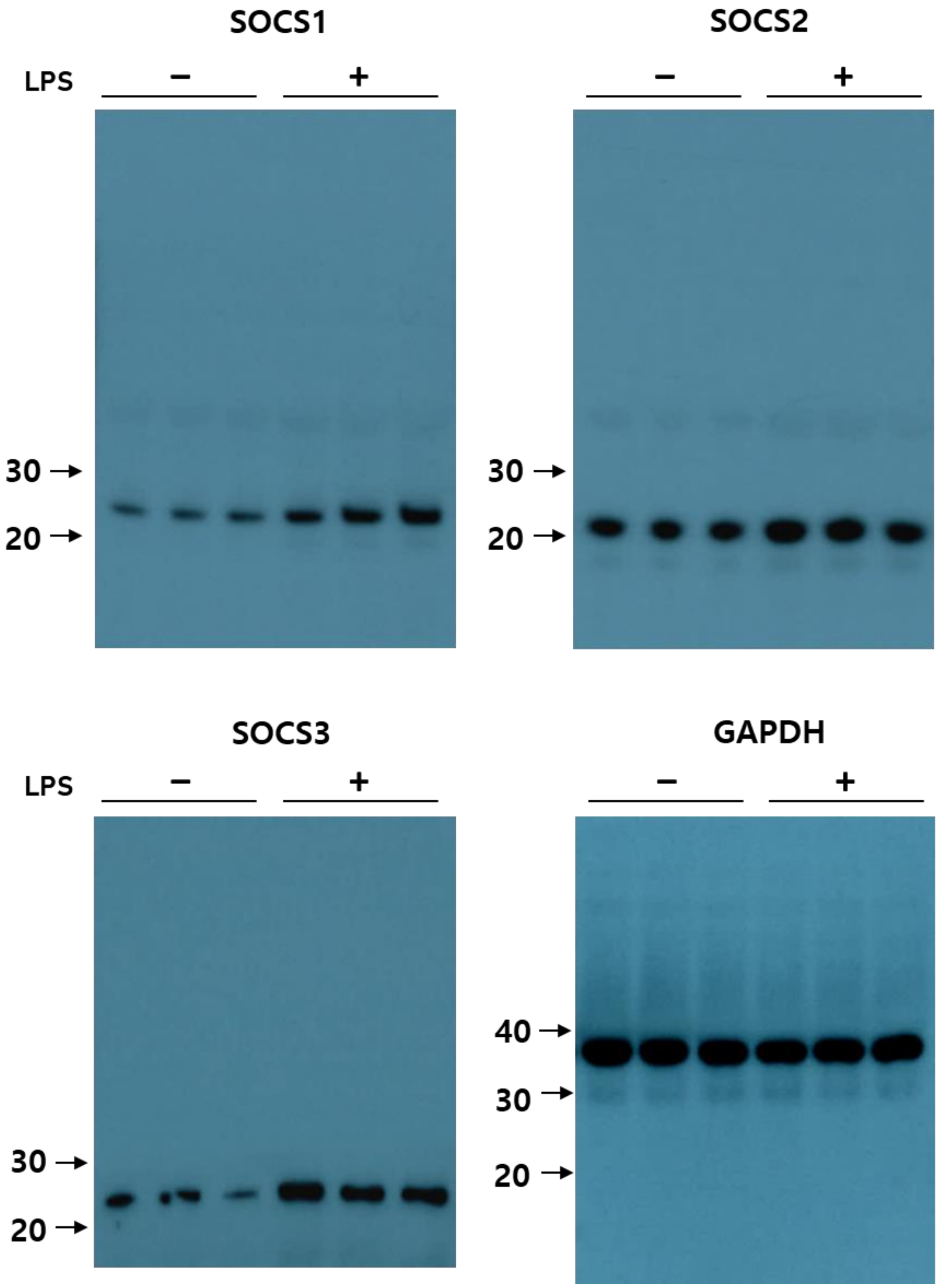

Supplementary figure S1. Full-length Western blots of SOCS1–3 and GAPDH, shown in Figure 1A.

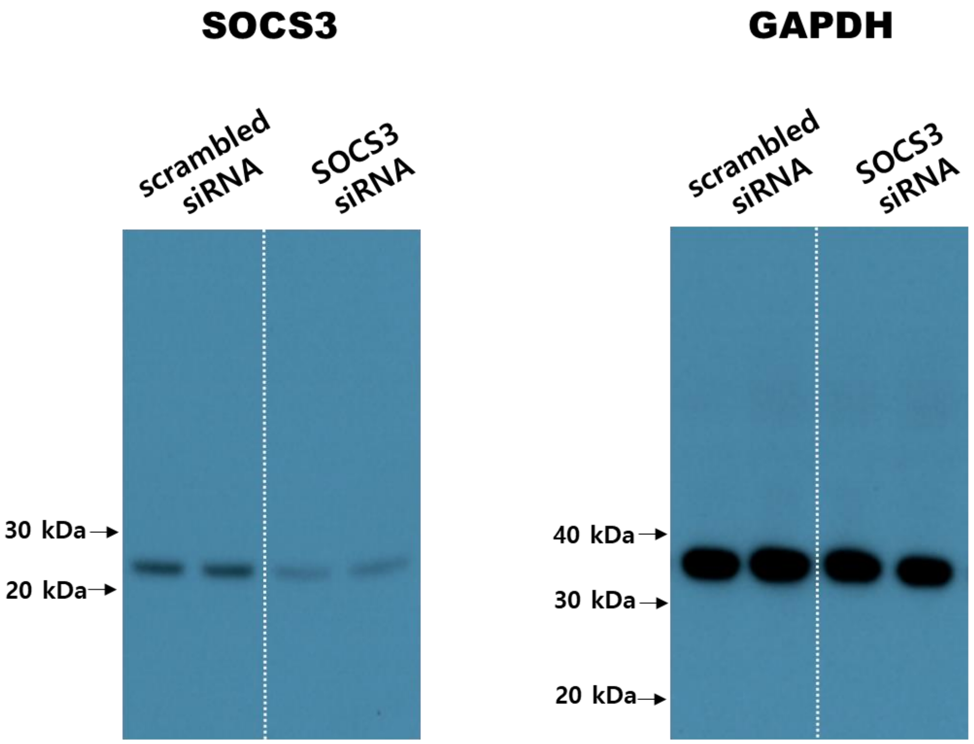

Supplementary figure S2. Full-length Western blots of SOCS3 and GAPDH in MSC transfected with scrambled (control) siRNA and SOCS3 siRNA, respectively.
